# Galanin Receptors in the Central Nervous System: Exploring Ligand Interactions, Signal Transduction, and Potential Clinical Implications

**DOI:** 10.3390/molecules31050792

**Published:** 2026-02-27

**Authors:** Anna Owczarek, Kamilla Blecharz-Klin

**Affiliations:** Department of Experimental and Clinical Pharmacology, Centre for Preclinical Research and Technology CePT, Medical University of Warsaw, Banacha 1B, 02-097 Warsaw, Poland

**Keywords:** galanin, neuropeptide, galanin receptor, CNS, spexin, neuropeptide signaling, GALP, therapeutic targets

## Abstract

Galanin is a highly conserved neuropeptide widely expressed in the central nervous system (CNS), where it regulates neurotransmission, neuroplasticity, and neuroendocrine functions. Its effects are mediated through three G protein-coupled galanin receptor subtypes, GalR1, GalR2, and GalR3, each exhibiting distinct tissue distributions, ligand affinities, and intracellular signaling mechanisms. Endogenous ligands, including galanin, galanin-like peptide (GALP), and spexin, interact with these receptors to trigger receptor-specific pathways, such as adenylyl cyclase (AC) inhibition (GalR1/GalR3) and phospholipase C-mediated calcium signaling (GalR2), enabling modulation of neuronal excitability, neurotransmitter release, and cell survival. Exogenous ligands, including peptide analogs and non-peptide agonists, have further elucidated receptor function and highlighted opportunities for pharmacological intervention. Preclinical evidence demonstrates that targeting galanin receptors (GalRs) can influence mood, cognition, pain perception, epilepsy, metabolic regulation, and neuroprotection, suggesting therapeutic potential across diverse CNS disorders. By integrating knowledge of ligand–receptor interactions and downstream signaling, this review highlights the central role of GalRs in CNS physiology and their emerging relevance as targets for clinical applications.

## 1. Introduction

Galanin is a neuropeptide first identified over forty years ago following its isolation from the porcine intestine [1]. It consists of 29 amino acid residues (30 in humans), and its name derives from its N- and C-terminal amino acids, glycine and alanine, respectively. The amino acid sequence of galanin is highly conserved across species, showing nearly 90% similarity [2]. Galanin is widely expressed in the peripheral and central nervous systems and the neuroendocrine axis, where it coexists with classical neurotransmitters and modulates synaptic transmission [1,3].

Since its discovery, galanin has been implicated in diverse physiological and behavioral processes, including stress responses [4], nociception [5], appetite regulation [6,7], reproduction [4], and cognitive functions such as learning and memory [8,9]. While several recent reviews have summarized the general physiological and clinical roles of galanin, these accounts have largely emphasized receptor distribution and canonical signaling properties. In contrast, emerging evidence points to increasing functional complexity of galanin action in the CNS, driven by receptor subtype specificity, cellular context, and ligand-dependent signaling bias, highlighting the need for an updated integrative perspective.

Galanin exerts its effects through three high-affinity G protein-coupled receptors—GalR1, GalR2, and GalR3—which primarily couple to Gi/Go and Gq/11 proteins and regulate intracellular effectors such as K^+^ and Ca^2+^ channels and AC activity [10,11]. These receptor subtypes differ in regional distribution, ligand selectivity, and downstream signaling, indicating receptor-specific roles in neuronal excitability and synaptic plasticity [12]. Recent original studies further demonstrate that GalR signaling can differentially and sometimes divergently modulate the release of classical neurotransmitters, including glutamate, GABA, acetylcholine (ACh), and monoamines, challenging simplified one-receptor–one-function models.

Advances in molecular pharmacology, receptor signaling, and structural biology have provided new insight into ligand–receptor interactions and biased signaling mechanisms of GalRs. These developments have prompted a shift from descriptive mappings of receptor expression toward mechanistic questions concerning how receptor subtype, ligand bias, and cellular environment jointly shape functional outcomes. Such insights are particularly relevant to neurological and psychiatric disorders, including Alzheimer’s disease [13,14,15], epilepsy [16], depression [17,18,19], and addiction [17,20].

Accordingly, this review integrates recent original research on GalR signaling with a specific focus on receptor subtype-dependent and context-dependent modulation of neurotransmission in the CNS. By highlighting emerging conceptual frameworks, unresolved controversies, and mechanistic advances beyond prior reviews, this article aims to clarify how recent findings fundamentally extend current understanding and inform the development of targeted therapeutic strategies.

## 2. Galanin Receptors: Distribution, Structure, and Signaling Pathways

**Figure 1:**
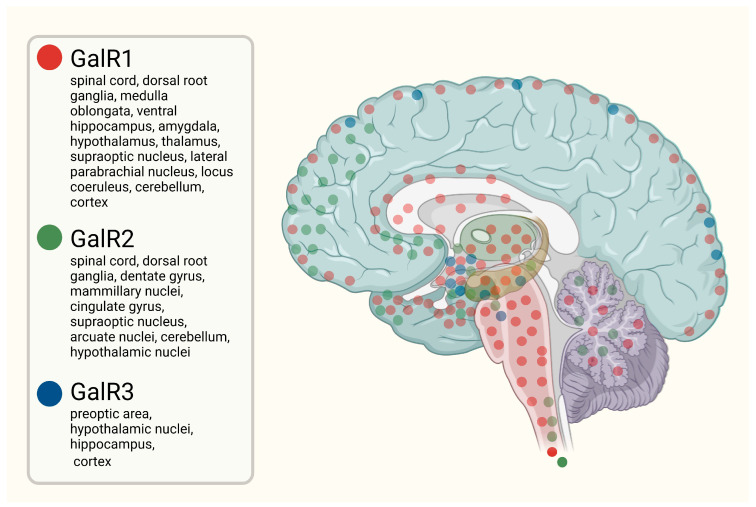
Simplified schematic representation of the distribution of galanin receptors (GalR1, GalR2, and GalR3) in the CNS. Created with BioRender.com.

Three GalR subtypes have been identified to date: GalR1, GalR2, and GalR3. All are class A seven-transmembrane G protein-coupled receptors sharing approximately 40–60% sequence homology [21]. GalR1 is predominantly expressed in the CNS, whereas GalR2 and GalR3 display broader distribution across the brain and peripheral tissues [22]. Despite structural similarities, these receptors differ markedly in ligand affinity, tissue distribution, intracellular signaling, and physiological function, indicating receptor subtype-specific roles in the central and peripheral nervous systems [21]. The distribution of GalRs within the CNS is illustrated in Figure 1.

**Figure 2:**
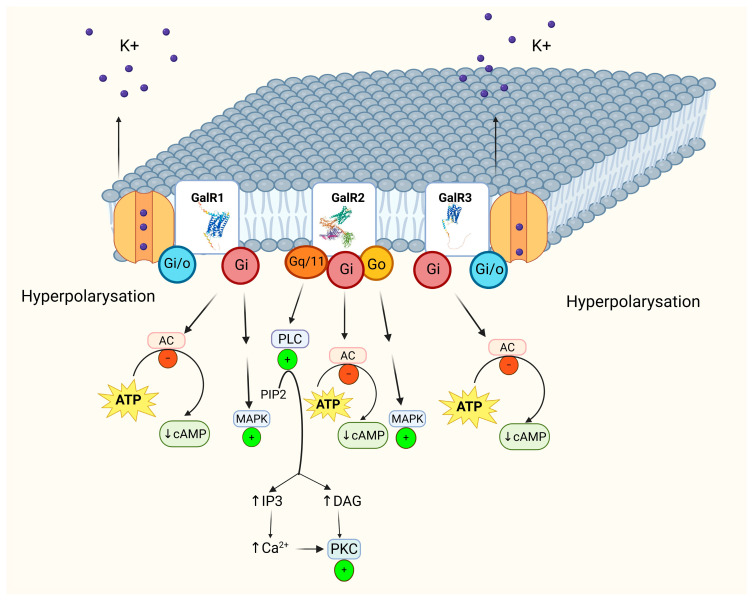
Galanin receptor subtypes (GalR1, GalR2, and GalR3) and their major intracellular signaling cascades in the CNS. Activation of GalR1 and GalR3, which are primarily coupled to Gi/Go proteins, results in inhibition of adenylyl cyclase (AC), reduced formation of cyclic adenosine monophosphate (cAMP) from adenosine triphosphate (ATP), and membrane hyperpolarization due to activation of potassium (K^+^) channels. GalR2 can interact with Gq/11, Gi and Go proteins, leading to activation of phospholipase C (PLC), which hydrolyzes phosphatidylinositol 4,5-bisphosphate (PIP2) into inositol 1,4,5-trisphosphate (IP3) and diacylglycerol (DAG). IP3 induces an increase in intracellular calcium ion (Ca^2+^) concentration, whereas DAG activates protein kinase C (PKC). All galanin receptor subtypes may also engage the mitogen-activated protein kinase (MAPK) signaling pathway (Based on: Iismaa and Shine 1999 [32]). Structures of galanin receptors were obtained from the Protein Data Bank and AlphaFold Database (PDB ID: 7XBD; DOI: 10.2210/pdb7XBD/pdb; PDB ID: 7XJJ; DOI: 10.2210/pdb7XJJ/pdb; the GALR3 model was obtained from the AlphaFold DB computed structure). Created with BioRender.com.

The physiological effects of galanin signaling in the brain, particularly in the hippocampus, are predominantly inhibitory [23]. GalRs exert their actions by coupling to distinct G proteins: GalR1 and GalR3 primarily signal via Gi/o proteins, leading to inhibition of AC, whereas GalR2 preferentially couples to Gq/11 proteins, activating phospholipase C and intracellular Ca^2+^ signaling pathways [22,24]. The major intracellular signaling cascades activated by individual GalR subtypes in the CNS are summarized in Figure 2. Recent structural studies revealed that galanin activates its receptors through an atypical, allosteric-like binding mode and that G protein selectivity is largely determined by intracellular loop 2 (ICL2). Notably, the ICL2 of GalR2 is sufficient to confer Gq coupling even when transplanted into receptors normally coupled to Gi or Gs, providing a molecular basis for receptor-specific and context-dependent signaling in the CNS [25].

Accumulating evidence indicates that GalRs can form heteromeric complexes with other G protein-coupled receptors (GPCRs), profoundly modulating their signaling. These heteromers can alter G protein subtype preferences, modify ligand pharmacology, and generate signaling responses unavailable to monomeric receptors. Notable examples include GalR1–5-HT1A heterodimers, which modulate serotonergic pathways in the limbic system, and GalR1–GalR2 complexes, in which distinct galanin fragments differentially activate signaling depending on the heteromeric context, highlighting their potential therapeutic relevance in mood regulation and addiction [25,26].

GalR1, the first identified galanin receptor, was isolated from the human Bowes melanoma cell line, a widely used human melanoma model, and mapped to chromosome 18q23 [21,27]. The human receptor consists of 349 amino acids and shares 92% sequence similarity with its rat homolog [28]. GalR1 is highly expressed in the spinal cord, pancreas, small intestine, and brain regions including the ventral hippocampus, amygdala, hypothalamus, thalamus, and locus coeruleus [21]. Activation of GalR1 inhibits AC, reduces intracellular cAMP levels, alters CREB phosphorylation, and promotes opening of G-protein-coupled inwardly rectifying K^+^ channels, resulting in neuronal hyperpolarization and reduced neurotransmitter release [19,21,29]. GalR1 signaling also engages MAP kinase pathways and suppresses protein kinase A activity, particularly in the central amygdala, contributing to antinociceptive effects following nerve injury [30]. Functional studies in GalR1-deficient mice further implicate this receptor in neuroendocrine regulation and seizure control [31].

In addition, GalR1-preferring agonists produce antinociceptive effects in rodent models, highlighting therapeutic potential in neuropathic pain [33]. Reduced GalR1 expression has also been associated with increased invasiveness of non-functioning pituitary neuroendocrine tumors [34].

GalR2 was identified in 1997 and localized to chromosome 17q25.3 [27]. The human GalR2 receptor consists of 387 amino acids and shares approximately 85% sequence similarity with the rat homolog [21,35]. Galanin binds GalR2 with high affinity (KD approximately 0.3 nM) [36]. GalR2 mRNA is widely distributed in the brain, including the hippocampus, cerebellar cortex, cingulate gyrus, mammillary nuclei, and hypothalamic regions, with particularly high expression in dorsal root ganglia. GalR2 is also expressed in peripheral tissues such as the small intestine, heart, kidney, and liver [36,37]. Functionally, GalR2 activation promotes phosphoinositide hydrolysis, IP_3_ generation, and intracellular Ca^2+^ elevation, leading to activation of CaMKII, MAPK, and calcium-dependent Cl^−^ channels [22,38]. Depending on cellular context, GalR2 may also inhibit cAMP production via Gi/o proteins and activate MAP kinase signaling through Go-mediated pathways, highlighting its signaling versatility [21]. GalR2 mediates pronociceptive and inflammatory pain responses by increasing the excitability of capsaicin-sensitive primary afferent neurons, likely via modulation of TRPV1 channels [33,39,40]. Upregulation of GalR2 expression during inflammation further supports its role in inflammatory pain signaling [41]. Elevated GalR2 expression in the human hippocampus suggests involvement in cognitive regulation and Alzheimer’s disease-related pathology [42].

GalR3, the most recently identified receptor subtype, was mapped to chromosome 22q13.1 and shares approximately 90% sequence identity with the rat receptor [21,27,43]. GalR3 exhibits lower ligand affinity than GalR2 (KD~75 nM) and displays a distinct pharmacological profile requiring amino acids 17–19 of galanin for ligand recognition [36,37]. In the brain, GalR3 expression is relatively restricted to hypothalamic and preoptic regions, including the paraventricular, ventromedial, and dorsomedial nuclei [44]. Peripheral expression has been detected in the pancreas, adrenal gland, spleen, and human testis [36,43]. Through Gi/o-mediated signaling, GalR3 contributes to neuronal hyperpolarization and inhibition of neurotransmitter release [21]. Its expression in pancreatic tissue suggests a role in insulin secretion and glucose homeostasis, while hypothalamic localization implicates GalR3 in appetite and fluid balance regulation [21]. Importantly, GalR3 mediates galanin-induced proliferation of hippocampal progenitor cells, identifying this receptor as a key regulator of adult neurogenesis and a potential contributor to the antidepressant effects of galanin [45].

## 3. Endogenous Galanin Receptor Ligands

Endogenous galanin acts as an agonist for all three receptor subtypes. The first 14 amino acids of galanin at the N-terminus are completely conserved across species [46]. The N-terminal sequence of galanin is essential for its interaction with receptors, as the galanin (1–16) fragment maintains the high binding affinity observed for the complete peptide [47]. In contrast to most neuropeptide agonists of class A GPCRs, which bind nearly perpendicular to the membrane plane, galanin binds almost parallel to the membrane plane of GalRs [22].

Following the identification of galanin in 1983 [1], several related peptides have been reported to be part of the galanin family. One of these, galanin-message-associated peptide (GMAP), derived from the same prepropeptide, was characterized. GMAP is derived from the C-terminal region of the galanin precursor and has been reported to mediate the spinal flexor reflex in rats and to possess antifungal properties [48]. GMAP shows very low or no binding affinity for GalRs, suggesting its effects may be mediated through alternative mechanisms [47]. Subsequently, galanin-like peptide (GALP), encoded by a different gene, was identified. GALP acts as a high-affinity agonist for both GalR1 and GalR2, with a marginally (18-fold) higher affinity for GalR2 [46]. More recently, a splice variant of GALP, alarin, has been discovered [48]. GMAP and alarin belong to the galanin peptide family but do not function as ligands for GalR1-3. Alarin is a 25-amino-acid peptide that appears to be expressed primarily in the CNS and peripheral tissues, similar to GALP. However, alarin has not yet shown detectable affinity for any of the three known GalR subtypes [47], suggesting that it may exert its biological effects through distinct, as-yet-unidentified receptors or signaling mechanisms. Emerging evidence indicates that alarin may play a role in regulating energy homeostasis, reproductive function, and vascular responses, although its precise physiological and pathophysiological functions remain largely unexplored [47,49].

Spexin (SPX), also known as neuropeptide Q, is an endogenous ligand of GalR2 and GalR3 [50]. Human GalR2 can be activated by both SPX and galanin, but it binds galanin more strongly. In contrast, human GalR3 is more selective for SPX [51]. It has been shown that spexin and galanin induce distinct active conformations of the GalR2, suggesting that these two ligands trigger different signaling pathways through the same receptor [50]. Research on hydrophobic molecular surface of fish SPX suggested that Lys11 being the only charged residue is believed to play a key role in receptor activation [51].

Spexin is composed of 14 amino acids [52] and the structural organization of the coding sequence of this neuropeptide is well conserved across species [50]. Moreover, the N-terminal sequence (1–16) is highly conserved in both GALP and SPX, while the remaining part of the sequence shows less conservation between the two [53]. SPX is highly expressed in the hypothalamus where it plays a crucial role in energy homeostasis and reproduction [52]. Furthermore, in humans, SPX mRNA is found in various tissues, including the skin, lungs, stomach, small intestine, colon, liver, pancreas, thyroid, adrenal glands, adipose tissue, and kidneys [50,51]. By acting on GalRs, SPX exerts various effects including the suppression of food intake and lipid absorption, a reduction in body weight, regulation of glucose balance, as well as the promotion of gastrointestinal motility [50,54]. SPX appears to mediate its hypophagic effect primarily through GalR3 [55]. On the other hand, by binding to GalR2, SPX can protect against insulin resistance by increasing glucose consumption in skeletal muscles. SPX may therefore represent a novel therapeutic target for the treatment of hyperglycemia and insulin resistance [56]. Although the exact role of SPX in reproduction is not yet fully understood, evidence suggests that neurons expressing gonadotropin-releasing hormone (GnRH) may both produce and respond to spexin [52]. Characteristics of endogenous galanin receptor peptide ligands are presented in Table 1.

## 4. Exogenous Ligands of the Galanin Receptors

Following the identification of galanin, several molecules acting on GalRs have been synthetized. These ligands were developed by conjugating mammalian galanin (1–13) to other bioactive molecules [57]. Despite extensive speculation regarding potential models of ligand–receptor interactions for chimeric GalR ligands, the mechanism determining why some ligands act as antagonists while others, sharing the same N-terminal galanin (1–13) recognition sequence, function as agonists remains unclear [58].

High-affinity chimeric peptide antagonists of GalRs include M15, M32, M35, M38, M40, and C7 [46,57]. While M38 and M40 only weakly or partially antagonize the effects of galanin, the antagonistic potencies of M32 and C7 are higher and comparable to those of M15 and M35 [59]. M15, also called galantide, was the first identified chimeric peptide antagonist of the GalRs. In the construct of M15, the galanin (1–13) fragment was coupled to the C-terminal fragment of substance P (residues 5–11). M15 exhibited approximately tenfold higher affinity than endogenous galanin for unspecified GalR subtypes in membrane preparations from rat tissues [47]. M15 has been shown to inhibit galanin-induced ACh release in the rat striatum and the hyperpolarization of locus coeruleus neurons [60,61]. Furthermore, the galanin-mediated inhibition of insulin release from pancreatic islets was completely reversed by galantide [61]. M32 consists of galanin (1–13), Neuropeptide Y (25–36), and amide. In a study by Xu et al., M32 exhibited the highest affinity reported to date for any GalR [59]. M40 (galanin [1-13]-Pro-Pro-[Ala-Leu]2-Ala amide) functions as a potent GalR antagonist in the brain, where it blocks galanin-induced feeding and reverses galanin-mediated inhibition of ACh release in the ventral hippocampus [62]. In contrast, it shows weak agonistic activity in peripheral tissues such as pancreatic islets and only weak antagonism in the spinal flexor reflex model [63]. C7 contains the N-terminal galanin (1–13), attached to the substance P antagonist, spantide, at the C-terminus. In vivo, both M40 and C7 exhibit antagonistic effects at doses equimolar to or lower than those of active galanin. What is more, compared with other chimeric galanin antagonists, C7 and M40 possess higher oxidative stability, representing a key advantage of these peptides [62].

M15, M32, M35, M38 and M40 have been valuable tools in galanin research but are limited by their relative non-specificity toward the different GalRs and by their weak interactions with other receptors [47]. The main limitations of using peptide ligands targeting GalR1-3 are their poor bioavailability in the CNS following systemic administration, due to their vulnerability to enzymatic degradation. In addition, these peptides have very short half-lives, lasting only a few minutes after intravenous injection [46,64].

Two non-peptide ligand agonists have been identified. The first of them, galnon, was developed in 2002 through screening of a combinatorial peptidomimetic library. It acts as a nonselective GalRs agonist, mediating its effects through inhibition of AC [65] and has been evaluated in models of anxiety, depression, feeding, and pain [47]. Saar et al. observed that galnon exhibits anticonvulsant effects, as intraperitoneal (i.p.) administration reduced the severity and delayed the onset of induced seizures in mice, while intrahippocampal injection shortened self-sustaining status epilepticus in rats. Anticonvulsant effects of galnon are primarily mediated through GalR1, as pretreatment with an antisense oligonucleotide targeting GalR1 mRNA resulted in the loss of anticonvulsant activity [64]. Furthermore, in a study by Wu et al., systemic administration of galnon alleviated heat hyperalgesia in rats with partial sciatic nerve injury in a dose-dependent manner [66]. Moreover, Lu et al. found that direct activation of GalRs in rats by galnon produced an antidepressant-like effect in the forced swim test [67]. In a study by Abramov et al., intracerebroventricular (ICV) and i.p. administration of galnon produced a pronounced, dose-dependent decrease in food intake in rodents. These findings indicate that galnon is a valuable compound for investigating the role of galanin in feeding regulation and related behavioral processes [68]. The non-peptide character of galnon gives the substance several advantages over ordinary peptide ligands, including higher stability and the ability to be administered systemically as galnon is capable of passing the blood–brain barrier (BBB) [65].

Galmic is another non-peptide agonist of GalRs with a higher affinity for GalR1 than for GalR2. Similar to galanin, it suppresses long-term potentiation in the dentate gyrus and effectively blocks sustained seizure activity following intrahippocampal or i.p. administration. Based on the study by Bartfai et al. galmic attenuates experimental status epilepticus in a dose-dependent manner. In the aforementioned study male adult Wistar rats underwent 30 min of perforant path stimulation (PPS) through a stimulating electrode to induce self-sustaining status epilepticus (SSSE). When injected into the dentate gyrus, shortly after seizure induction, low doses of galmic reduced seizure duration, while higher doses completely abolished seizures. Intraperitoneal administration was also effective at 2 mg/kg, significantly reducing seizure severity and duration, though lower doses (1 mg/kg) had no effect [69]. Despite these promising results, it seems that the therapeutic potential of both galnon and galmic is limited due to their low affinity and lack of selectivity, as they also interact with other receptors such as dopamine (DA) and melanocortin receptors, leading to off-target physiological effects [46,47].

To date, only two non-peptide GalR antagonists have been described, spirocoumaranon (Sch 202596) and 2,3-dihydro-2-(4-methylphenyl)-1,4-dithiepin-1,1,4,4-tetroxid, that both exhibit low binding affinity. In contrast to galnon and galmic, these compounds have not been tested in vitro or in vivo model systems [46].

Characteristics of exogenous GalR chimeric peptide ligands are presented in Table 2. Most chimeric M-series peptides act as non-selective GalR antagonists with limited subtype specificity. Functional selectivity may vary depending on experimental system. Features of exogenous non-peptide ligands for GalRs are summarized in Table 3.

## 5. Physiological Role of Galanin and Galanin Receptors in the CNS

### 5.1. Regulation of Neurotransmission and Neuroplasticity

Galanin is a important regulator of neurotransmission in both the central and peripheral nervous systems, modulating the release of classical neurotransmitters [2,24]. In the hippocampus, galanin exerts region-specific effects on ACh release, decreasing it in the ventral hippocampus while increasing it in the dorsal hippocampus [70]. The ability of galanin to suppress cholinergic activity, together with hyperinnervation of galanin fibers in the basal forebrain of Alzheimer’s disease patients, suggests its contribution to cholinergic dysfunction in this disorder [2].

Galanin predominantly inhibits noradrenaline (NA) and serotonin (5-HT) neurons. In noradrenergic neurons, it activates GPCR-linked K^+^ channels, suppressing neuronal firing and enhancing auto-inhibition via α2 receptors. Galanin reduces 5-HT neuron activity mainly via GalR1 and GalR3, whereas GalR2 activation stimulate 5-HT release [71]. Lateral ventricle injection of galanin caused a prolonged decrease in 5-HT release in the ventral hippocampus in vivo [70].

**Figure 3:**
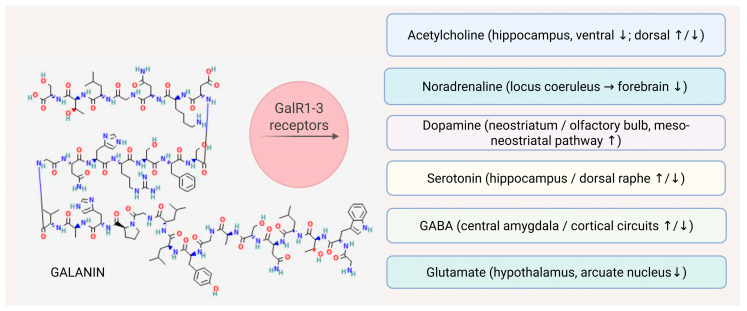
Effect of galanin on neurotransmitter levels and signaling pathways in the CNS. Galanin acts as a neuromodulator in the CNS by activating three G-protein-coupled receptors (GALR1–3), leading to inhibition of excitatory neurotransmitter release, modulation of inhibitory signaling, and regulation of monoaminergic and cholinergic systems. These effects contribute to network stabilization, neuroprotection, and context-dependent regulation of cognition and emotional behavior. ↓—inhibitory/decreased release; ↑—facilitatory/increased release; ↑/↓—context-dependent modulation. Structure of Galanin. Image from National Center for Biotechnology Information [73]. PubChem Compound Summary for CID 16133823, Galanin. Retrieved 29 December 2025, from https://pubchem.ncbi.nlm.nih.gov/compound/Galanin. Created with BioRender.com.

Conversely, galanin enhances DA synthesis in the forebrain, increasing L-DOPA accumulation in the neostriatum and olfactory bulb via tyrosine hydroxylase activation, without affecting NA-rich regions such as the neocortex or ventral hippocampus. This dopaminergic effect occurs at the soma-dendritic level of the meso-neostriatal pathway and is accompanied by a dose-dependent decrease in spontaneous locomotor activity [72]. (Figure 3).

Galanin also modulates neuroendocrine functions, increasing growth hormone, prolactin, and luteinizing hormone, while inhibiting glucose-stimulated insulin secretion and regulating gastrointestinal motility. Its expression is upregulated by estrogen, neuronal activation, denervation, or nerve injury [2]. Acting as an immunomodulatory neuropeptide, galanin influences neuroplasticity by modulating immune cell sensitivity and attenuating neurogenic inflammation; GalR3 activation reduces swelling and vascular leakage in certain autoimmune conditions [74]. Following nervous system injury, GalR2-mediated signaling reduces excitotoxic neuronal death, indicating a neuroprotective role [75].

### 5.2. Regulation of Mood, Stress, and Reward

Central galanin signaling profoundly influences mood and stress, as evidenced by rodent experiments demonstrating potent anxiolytic effects, highlighting its central role in anxiety regulation [76,77,78,79,80]. However, the effects of galanin largely depend on the infusion site [78]. For example, injecting galanin into the amygdala caused increased anxiety in rats in a punished drinking test. However, it produced no anxiety-related effects in the elevated plus-maze or when injected into the parietal cortex, suggesting that galanin’s anxiogenic actions depend on both brain region and behavioral test [79]. Galanin injected in the dorsal hippocampus of rats was found to increase anxiety-like behavior, while blocking GalR2 produced anxiolytic effects. Pretreatment with the GalR2 antagonist M871 prevented the anxiogenic effect of galanin, indicating that GalR2 mediate this response. Overall, the findings suggest that galanin exerts both pharmacological and tonic anxiogenic actions in the dorsal hippocampus through GalR2 activation [80]. Furthermore, the study by Holmes et al. found that mice lacking the GalR1 displayed increased anxiety-like behavior specifically on the elevated plus maze, while their performance on other anxiety tests and general neurological function remained normal. These results support the idea that galanin exerts anxiolytic effects also through GalR1, particularly under high-stress conditions [81].

Like several other neuropeptides, galanin can modify neural activity in brain regions that are crucial for stress-related behaviors [82]. In a study by Zhao et al. on mice, the mRNA levels of galanin and GalR1 were enhanced following stress, while fluoxetine reversed the behavioral changes by reducing galanin expression and upregulating GalR2 and α2A-adrenergic receptor levels, suggesting that activating the galanin system, with corresponding changes to noradrenergic systems, following chronic stress may modulate stress-related behaviors [76]. Mitsukawa et al. found out that ICV injected galanin modulates stress response in mice in a bidirectional, dose-dependent manner: a high dose reduced restraint stress-induced hyperthermia, while a low dose slightly increased it, and both doses affected stress hormone levels and locomotor activity. These bidirectional effects were absent in GalR1 knockout mice, highlighting that GalR1 is essential for galanin’s modulation of stress response [83].

There is considerable evidence that galanin plays an important role in reward processing and addictive behaviors [17,24]. Drugs targeting GalRs can influence both responses to addictive substances and stress-related behaviors. Stress- and drug-related behaviors are closely linked, as stress can trigger drug-seeking, while drug use and withdrawal can activate brain circuits that mediate the stress response [82]. Genetic analysis revealed that GalR3, but not GalR1 or GalR2, is significantly associated with alcoholism due to a single nucleotide polymorphism. Furthermore, combining the GalR3 risk allele with GAL haplotypes or diplotypes modestly increased the risk for alcoholism, suggesting that GalR3 mediates galanin’s effects on alcohol-related behaviors [84]. GalR1 appears to modulate opiate and nicotine dependence, as GalR1 knockout mice show more severe withdrawal symptoms, whereas GalR2 and GalR3 are less involved in these effects. Overall, GalRs are important mediators of addiction-related behaviors, making them potential pharmacological targets for treating alcohol, opiate, and nicotine dependence [85].

### 5.3. Modulation of Pain and Sensory Processing

One of the most frequently suggested physiological function of galanin is pain modulation at the level of the spinal cord [86]. Galanin is expressed in the dorsal root ganglia and spinal cord where it plays a complex role in pain modulation, showing both inhibitory and facilitatory actions. Using two transgenic mouse lines that overexpress galanin in dorsal root ganglia neurons, Holmes et al. demonstrated that galanin exerts predominantly inhibitory effects on nociception, increasing pain thresholds in intact animals and reducing mechanical allodynia after nerve injury [87]. It has been proposed that GalR1-driven hyperpolarization of sensory neurons and interneurons underlies both its analgesic properties and its synergy with opioid drugs, with GalR1 agonists thought to inhibit glutamate release in the spinal cord. In contrast, the depolarizing actions mediated by GalR2, although beneficial for neural regeneration, may enhance pain signaling [85]. In addition, galanin plays an excitatory, pro-nociceptive role in inflammatory pain by enhancing capsaicin-evoked activity in peripheral sensory neurons through GalR2-mediated modulation of VR1-positive nociceptors [41].

Galanin produces antinociception in neuropathic rats through activation of GalRs in the nucleus accumbens, with GalR1 specifically implicated due to its upregulated expression after peripheral nerve injury. The reversal of galanin’s effect by a non-selective GalR antagonist galantide supports that galanin-mediated signaling is a key contributor to the enhanced antinociceptive response after nerve injury [88]. Mice deficient in GalR1 display heightened baseline pain sensitivity and develop longer-lasting mechanical and thermal hypersensitivity after sciatic nerve injury compared with wild-type animals. These results further support that signaling through GalR1 normally provides an inhibitory influence on nociception and helps limit neuropathic pain after peripheral nerve damage [89].

The development of GalR ligands in recent years has strengthened the possibility of targeting the galanin system for new analgesic therapies [90].

### 5.4. Involvement in Learning, Memory, and Cognitive Function

The galaninergic system contributes to the regulation of learning and memory [8]. Studies in rodents suggest that central administration of galanin (mainly intraventricularly) plays an inhibitory role in hippocampal-dependent learning [91]. The coexistence of galanin with ACh in the rat septohippocampal pathway suggests that it may help support the function of cholinergic system and influence learning and memory. Galanin has been shown to impair performance in several rodent learning and memory tasks. Rats given daily intraventricular injections of galanin learned the position of underwater platform in Morris water maze more slowly [92]. It is worth mentioning, that galanin injected into the medial septum/diagonal band of Broca (MS/dBB), in rats, did not impair, and in some doses even tended to facilitate, spatial learning, suggesting a facilitatory role of septal galanin on hippocampal-dependent cognition. However, when combined with the muscarinic antagonist scopolamine, galanin caused marked learning impairments and excessive ACh release, indicating that optimal muscarinic cholinergic activity is critical for galanin’s effects on memory and cognitive function [93].

Galanin overexpression appears to impair learning and memory, as GAL-transgenic mice show deficits in spatial, olfactory, and trace fear conditioning tasks, likely due to galanin’s inhibition of ACh, glutamate, 5-HT, and NA release in the hippocampus. These findings link galanin overexpression to cognitive impairments relevant to Alzheimer’s disease. GalR1 knockout mice perform normally on most memory tasks but show deficits in trace cued fear conditioning, suggesting GalR1 selectively contributes to certain hippocampus-dependent aversive memories, while other receptor subtypes may mediate spatial and social memory [94]. Galanin injected into the dorsal and ventral dentate gyrus of the hippocampus, regions rich in GalR2, impaired spatial learning in the Morris water maze, an effect blocked by the galanin antagonist M35. In contrast, galanin infusions into ventral CA1 or ventral CA3, which mainly express GalR1, did not affect learning, suggesting that galanin influences spatial learning primarily through GalR2 in noradrenergic-rich hippocampal regions [95].

### 5.5. Role in Appetite Control and Energy Homeostasis

Galanin is undoubtedly involved in the regulation of food intake and body weight [96,97]. Injection of galanin into the hypothalamus, especially the paraventricular nucleus, promotes food intake in rats [6]. As presented by Zhang et al. galanin injections (i.p.) did not affect food intake, but ICV administration significantly increased food intake.

Co-injection of galanin and spexin demonstrated that spexin counteracted galanin-induced feeding and modulated hypothalamic appetite-related gene expression, indicating opposing anorexigenic and orexigenic actions of these peptides [97]. A rapid rise in central galanin levels can increase overall feeding and preferential fat consumption, an effect that can be inhibited by ICV administration of the galanin antagonists M40 and C7 [6]. A study by Saar et al. demonstrated that, unlike the GalR1-selective agonist M617, which robustly increases high-fat food intake in rats, novel GalR2-selective agonists do not stimulate consumption, supporting a primary role for GalR1 in galanin-induced feeding [98]. Galanin does not alter water intake but preferentially promotes fat consumption, as evidenced by reduced fat intake in galanin knockout mice, partially rescued by peptide replacement [29,99,100]. Consistent with these metabolic effects, GalRs activate AKT/MAPK signaling pathways that regulate adipogenesis, apoptosis, and cell proliferation [29].

Galanin appears to enhance insulin sensitivity in type 2 diabetic rats through GalR2, via promoting glucose transporter 4 (GLUT4) expression and translocation, as evidenced by its effects being reversed by a GalR2 antagonist and mimicked by a GalR2 agonist [101].

A study by Baranowska et al. reported elevated plasma galanin levels in obese women, while levels in women with anorexia nervosa were comparable to controls, suggesting an association between galanin dysregulation and obesity-related body weight status or pathogenesis [102].

The functional roles of galanin receptor subtypes in physiological and pathological conditions are summarized in Table 4.

## 6. Potential Clinical Implications and Therapeutic Potential of Targeting Galanin Receptors

Imbalance in galanin has been linked to a range of neuroendocrine, neurological and psychiatric disorders, including pain perception, Alzheimer’s disease, seizures, eating disorders, alcoholism, diabetes, and spinal cord conditions [48]. These findings highlight the potential for targeted therapies at GalRs as a promising approach for treating these diverse conditions.

Galanin is deeply involved in mood regulation because it interacts with both 5-HT and NA systems, which are central to depression and its treatment. Evidence shows that galanin can inhibit 5-HT release and alter 5-HT1A receptor function, suggesting that dysregulated galanin signaling may contribute to depressive symptoms. Importantly, blocking GalR1 or GalR3, or activating GalR2, has produced antidepressant-like effects in rats, as demonstrated by Ögren et al. [103]. In a study by Swanson et al. on mice, rats and guinea pigs selective GalR3 antagonists (SNAP 37889 and SNAP 398299) produced anxiolytic effects, possibly by attenuating the inhibitory influence of galanin on 5-HT transmission at the level of the dorsal raphe nucleus. Overall, the findings indicate that blocking GalR3 may alleviate anxiety and depression by reducing galanin’s inhibitory influence on 5-HT transmission. Hence, targeting GalR3 could offer new therapeutic options for patients who do not respond well to current antidepressant drugs [104]. On the other hand, the study by Saar et al. developed novel systemically active galanin analogs with GalR2 selectivity and chemical modifications that enhance brain penetration upon intravenous injection. Among them, peptide J18 demonstrated antidepressant-like effects in mouse models of depression, including the forced swim test and tail suspension test, via GalR2 activation, highlighting GalR2 agonists as promising drugs for depression [105].

Preclinical studies increasingly suggest that galanin and its receptors are promising targets for anxiolytic and antidepressant therapies, positioning the galaninergic system as a key focus for future drug development [103,106]. Knockout mouse studies, changes in galanin expression after antidepressant therapy support the involvement of multiple GalR subtypes in mood regulation. Nevertheless, a study by Barrera et al. shows that although yohimbine-induced activation of the NA system triggers galanin release in the central amygdala and produces anxiolytic effects, this galanin is not co-released from noradrenergic terminals but instead originates from nearby galaninergic neurons activated indirectly by increased NA activity. These findings indicate that galanin’s role in anxiety is highly region, pathway-, and context-dependent, posing significant challenges for developing galanin-targeted anxiolytic or antidepressant therapies [107].

Galanin’s ability to inhibit Ach release, along with the observed overabundance of galanin fibers in the basal forebrain of individuals with Alzheimer’s disease, points to a potential involvement of galanin in the cholinergic deficits associated with that condition [2]. Galanin impairs learning and memory in rats, mainly by acting in the ventral hippocampus and medial septum, likely through inhibition of ACh signaling. GalR agonists like M40 can block these effects and, when combined with cholinergic agonists, improve memory [91]. Based on a study by Crawley et al., intraventricular administration of galanin inhibits ACh release in the rat ventral hippocampus and produces deficits in learning and memory tasks. In Alzheimer’s disease, galanin is overexpressed, hence blocking its receptors may enhance cholinergic function, offering a potential therapeutic strategy for dementia [108]. A study by Arletti et al. demonstrated that galantide, a GalR antagonist, improves social memory in rats. When administered ICV, it significantly enhanced recognition of a familiar juvenile, without affecting general social investigation or being confounded by stress or reward [109]. These results suggest that blocking galanin, an inhibitory modulator of cholinergic neurons, can facilitate memory consolidation, indicating potential therapeutic value for memory impairments such as those seen in Alzheimer’s disease [13].

Peripheral galanin plays an important role in pain modulation, and the use of newly available receptor-selective ligands has clarified the distinct functions of GalR1 and GalR2 in nociception. Activation of peripheral GalR1 with the selective agonist M617 markedly reduces capsaicin-evoked nociceptive behaviors, whereas activation of GalR2 with AR-M1896 enhances these responses, an effect reversed by the GalR2 antagonist M871. These findings show that peripheral GalR1 mediates anti-nociception while GalR2 promotes pro-nociception, highlighting their potential as therapeutic targets in peripheral pain [39]. A targeted agonist for the GalR1 may represent a promising therapeutic approach for managing neuropathic pain [61]. Furthermore, studies showing that intraplantar injection of the GalR2 agonist M1896 enhanced mechanical and thermal pain responses following chronic constriction injury of the median nerve, whereas the GalR2 antagonist M871 had the opposite effect, suggesting that GalR2 antagonists may also have potential as analgesics [110].

By contributing to the regulation of excitation–inhibition balance in the brain, galanin may help mitigate epilepsy, with evidence showing that increasing its activity or receptor stimulation can lessen seizures and associated brain alterations [111].

Hippocampal galanin functions as an endogenous anticonvulsant during status epilepticus (SE), acting primarily through GalR1 and GalR2 [16]. It appears that centrally administered galanin has strong anticonvulsant effects, with the hippocampus being the most responsive site, while other regions like the caudate-putamen, substantia nigra, and nucleus accumbens require higher or specific doses, and the ventral tegmental area shows no effect, indicating that galanin’s seizure-suppressing actions vary depending on the targeted brain region [112]. The study by Mazarati et al. demonstrated that galanin depletion during SE may help sustain seizures, while galanin elevation aid seizure termination; exogenous galanin stopped or prevented SE through these receptors, whereas blocking them with antagonists M35, M40 and M15 facilitated seizure development. GalR2-preferring agonist (2-Ala-galanin) prevented, but could not halt ongoing SE [16]. On the other hand, GalR1 knockout mice experience more severe seizures and greater hippocampal injury, demonstrating that GalR1 normally protects against limbic status epilepticus-related damage [113]. This highlights GalR1 and GalR2 as key mediators of galanin’s anticonvulsant properties and underscores the potential of targeting these receptors with agonists for epilepsy therapy.

GalRs may also be attractive targets for the development of novel therapeutics for alcohol use and addiction. The study by Ash et al. demonstrated that the selective GalR3 antagonist (SNAP 37889) lowered operant responding for ethanol, sucrose, and saccharin in alcohol-preferring rats, without influencing their activity levels or anxiety-related behaviors. These results suggest that blocking GalR3 reduces alcohol intake and may play a role in regulating the rewarding effects of both natural and drug reinforcers [114].

As mentioned before, galanin improves insulin sensitivity in type 2 diabetic rats, by enhancing GLUT4 expression and translocation in adipose tissue, an effect that is blocked by a GalR2 antagonist and mimicked by a GalR2 agonist. These findings indicate that central GalR2 mediate galanin’s beneficial metabolic actions, supporting GalR2 agonists as potential treatments for insulin resistance and type 2 diabetes. On the other hand, since GalR1-selective agonist seem to increase high-fat food intake in rats it is possible that GalR1 antagonists may offer therapeutic potential for certain forms of obesity [98,100].

To date, no galanin receptor-targeted therapies have been approved for clinical use, and while early human studies (e.g., with the GALR3 antagonist HT-2157/SNAP-37889) were initiated, they were terminated due to safety concerns and have not progressed to later clinical phases, with no detailed clinical data publicly available.

Despite the therapeutic potential of GalRs, several challenges must be considered in translating these findings into clinically viable interventions. Receptor subtype redundancy may result in compensatory effects that diminish the efficacy of selective ligands, while the widespread expression of GalR1–3 across multiple tissues raises the risk of off-target actions. Achieving CNS-selective pharmacokinetics remains a key hurdle, particularly for peptide-based ligands, limiting their effective central delivery and therapeutic window. Recent advances, however, provide strategies to overcome these obstacles. Biased agonism allows preferential activation of beneficial signaling pathways while minimizing adverse effects. Peptide stabilization techniques, including cyclization and chemical modification, enhance in vivo half-life, and innovative delivery approaches—such as nanoparticle carriers or BBB-permeable formulations—may improve CNS selectivity. Integrating these strategies into preclinical and translational studies will be critical for realizing the therapeutic promise of GalR-targeted interventions.

## 7. Summary and Conclusions

Galanin receptors function in the CNS as a context-dependent modulatory system, where receptor subtype specificity, biased agonism, and cellular environment collectively shape diverse and sometimes divergent signaling outcomes. Recent evidence challenges the traditional one-receptor–one-function model and underscores unresolved roles in cognition, mood, stress, pain, and metabolic regulation. By integrating these insights, a mechanistic framework emerges in which receptor subtype, ligand bias, and cellular context jointly determine effects, reconciling conflicting observations across experimental systems.

Despite strong preclinical support, clinical translation remains limited by receptor redundancy, widespread tissue distribution, off-target effects, and challenges in CNS-selective delivery of peptide ligands. Future directions include single-cell mapping of receptor subtypes, in vivo dissection of biased signaling, and validation of receptor-selective ligands in disease-relevant models, which will be critical for realizing the therapeutic potential of GalR-targeted interventions.

## Figures and Tables

**Table 1:** Endogenous galanin family peptides and their relationship to galanin receptors.

Peptide	Type	Receptor Affinity and Selectivity	Key Features and Activity
**Galanin**	Endogenous peptide	GalR1-3 (agonist)	N-terminal 1–14 highly conserved; binds parallel to membrane plane, full peptide required for high affinity; regulates stress response, nociception, appetite, reproduction, learning and memory
**Galanin** **(1–15)**	N-terminal fragment of galanin	Preferential activation of GalR2 > GalR1, weak GalR3	Retains most biological activity of full-length galanin; stronger neurotrophic and excitatory effects; used experimentally as GalR2-biased ligand
**GMAP**	Endogenous peptide	Low/no affinity for GalR1-3	Derived from C-terminal of preprogalanin; mediates spinal flexor reflex, antifungal activity, effects likely via other mechanisms
**GALP**	Endogenous peptide	GalR1, GalR2 (agonist); higher affinity for GalR2	Encoded by a different gene; similar expression to alarin, N-terminal sequence 1–16 conserved with SPX
**Alarin**	Endogenous peptide (splice variant of GALP)	No detectable affinity for GalR1-3	25 amino acids; expressed in CNS and periphery, may act through unknown receptors; involved in energy homeostasis, reproduction, vascular responses
**Spexin**	Endogenous peptide	GalR2, GalR3 (agonist)	14 amino acids; N-terminal 1–16 conserved with GALP, regulates feeding, glucose homeostasis, reproduction; GalR3 mediates hypophagic effect; GalR2 protects against insulin resistance

**Table 2:** Exogenous (chimeric/synthetic) peptide ligands for galanin receptors.

Ligand	Type	Receptor Affinity and Selectivity	Key Features and Activity
**M15 (Galantide)**	Antagonist	High affinity for GalR1-3; nonselective	Galanin (1–13) + substance P fragment; inhibits galanin-induced effects; first chimeric antagonist; inhibits ACh release, insulin inhibition
**M32**	Antagonist	Highest reported affinity for any GalR; slightly higher for GalR1 > GalR2 > GalR3, but broadly active	Galanin (1–13) + NPY (25–36) + amide; potent tool for receptor studies
**M35**	Antagonist	Nonselective across GalR1-3	Similar activity to M15
**M38**	Antagonist	Weak/partial antagonist; minimal selectivity	Limited activity
**M40**	Antagonist	Potent antagonist in CNS; weak agonist peripherally, moderate selectivity for GalR1/GalR2	Galanin (1–13)-Pro-Pro-(Ala-Leu)2-Ala amide, blocks feeding, ACh release; high oxidative stability
**C7**	Antagonist	Potent antagonist at GalR1 and GalR2; limited data on GalR3	Galanin (1–13) + spantide; high oxidative stability; effective in vivo

**Table 3:** Exogenous non-peptide ligands for galanin receptors.

Ligand	Type	Receptor Affinity and Selectivity	Key Features and Activity
**Galnon**	Agonist	Nonselective agonist (GalR1-3)	Inhibits AC; crosses BBB; systemic administration; evaluated for seizures, depression, feeding; anticonvulsant mainly via GalR1
**Galmic**	Agonist	Higher affinity for GalR1 than GalR2	Suppresses Long-Term Potentiation (LTP), reduces seizures; systemic and intracerebral administration effective; limited selectivity
**Spirocoumaranon (Sch 202596)**	Antagonist	Low affinity, nonselective across GalR1-3	Not yet tested in vitro/in vivo
**2,3-Dihydro-2-(4-methylphenyl)-1,4-dithiepin-1,1,4,4-tetroxid**	Antagonist	Low affinity, nonselective across GalR1-3	Not yet tested in vitro/in vivo
**SNAP 37889**	Antagonist	GalR3-selective antagonist; minimal activity at GalR1/2	Small-molecule inhibitor; used to probe Gal3 function in stress, mood, and feeding studies; orally bioavailable; crosses BBB
**SNAP 398299**	Antagonist	GalR3-selective antagonist	Improved pharmacokinetics and receptor specificity; used for in vivo studies of GalR3-mediated behaviors; minimal off-target GalR1/2 activity
**AR-M1896**	Antagonist	GalR3-selective antagonist	Small molecule; inhibits GalR3-mediated signaling in vitro and in vivo; tool for studying GalR3 role in anxiety, depression, and metabolic regulation

**Table 4:** Comparison of galanin receptors: GalR1, GalR2, and GalR3.

Feature	GalR1	GalR2	GalR3
**Primary** **signaling** **pathway**	Gi/o (inhibitory; ↓ cAMP)	Gq/11 (excitatory; ↑ IP3/Ca^2+^)	Gi/o (inhibitory)
**Localization** **(brain &** **peripheral** **tissues)**	Dorsal root of spinal cord, ventral hippocampus, amygdala, hypothalamus, thalamus, supraoptic nucleus, lateral parabrachial nucleus, locus coeruleus; small intestine and pancreas	Broad CNS distribution: hippocampus (dentate gyrus), cerebellar cortex, mammillary nuclei, cingulate gyrus, posterior hypothalamus, supraoptic nucleus, arcuate nucleus; highest levels: dorsal root ganglia; also in small intestine, heart, kidney, liver	Restricted CNS distribution: preoptic area and hypothalamus (premammillary, paraventricular, ventromedial, dorsomedial nuclei); peripheral expression: testis, adrenal gland, spleen, pancreas
**Role in** **mood &** **stress**	Mediates anxiolytic effects in high-stress conditions; required for galanin’s bidirectional stress modulation	Mediates anxiogenic effects in dorsal hippocampus; blockade prevents anxiety-like behavior	Genetic variants linked to alcoholism; modulates addiction-related behaviors
**Role in** **addiction**	Modulates opiate and nicotine dependence	Less involved in drug-related behaviors	Strong association with alcoholism risk
**Role in pain** **modulation**	Major antinociceptive receptor; hyperpolarizes sensory neurons; ↓ neuropathic pain	Pronociceptive in inflammatory pain; ↑ capsaicin-evoked activity; supports neural regeneration	Less established; limited involvement
**Role in** **memory &** **learning**	Required for certain aversive memory tasks	Impairs spatial learning when activated in DG; major receptor mediating learning deficits	Less defined; not strongly linked to hippocampal memory
**Role in feed-** **ing, energy** **homeostasis,** **& metabolic** **regulation**	Primary receptor driving high-fat food intake	↑ insulin sensitivity via central activation; ↓ GLUT4 expression/translocation	Not strongly linked to appetite control

(↑ increase/activation, ↓ decrease/inhibition).

## Data Availability

No new data were created or analyzed in this study.

## Abbreviations

The following abbreviations are used in this manuscript:

5-HT	5-Hydroxytryptamine/serotonin
5-HT1A	5-Hydroxytryptamine receptor 1A
AC	adenylyl cyclase
ACh	acetylcholine
AKT	protein kinase B
ATP	adenosine triphosphate
BBB	blood–brain barrier
CA1	Cornu Ammonis area 1
CA3	Cornu Ammonis area 3
CAMKII	Calcium/Calmodulin-dependent Protein Kinase II
cAMP	adenosine 3′,5′-cyclic monophosphate
CNS	central nervous system
CREB	cAMP response element-binding protein
DA	dopamine
DAG	diacylglycerol
GALP	galanin-like peptide
GalR1	galanin receptor subtype 1
GalR2	galanin receptor subtype 2
GalR3	galanin receptor subtype 3
GalRs	galanin receptors
GLUT4	glucose transporter 4
GMAP	galanin-message-associated peptide
GnRH	gonadotropin-releasing hormone
GPCR	G protein-coupled receptor
ICL2	intracellular loop 2
ICV	intracerebroventricular
IGF-I	insulin-like growth factor-I
i.p.	intraperitoneal
IP3	inositol 1,4,5-trisphosphate
KD	dissociation constant
L-DOPA	L-3,4-dihydroxyphenylalanine
LTP	Long-Term Potentiation
MAP	Mitogen-Activated Protein
MAPK	Mitogen-Activated Protein Kinase
MS/dBB	medial septum/diagonal band of Broca
NA	noradrenaline
NFPitNETs	non-functioning pituitary neuroendocrine tumors
NPY	neuropeptide Y
PIP2	phosphatidylinositol 4,5-bisphosphate
PKA	protein kinase A
PKC	protein kinase C
PLC	phospholipase C
PPS	perforant path stimulation
SE	status epilepticus
SPX	spexin
SSSE	self-sustaining status epilepticus
TRPV1	transient receptor potential vanilloid type 1
VR1	vanilloid receptor 1
